# Characterization of Deqi Sensation and Acupuncture Effect

**DOI:** 10.1155/2013/319734

**Published:** 2013-06-20

**Authors:** Xing-Yue Yang, Guang-Xia Shi, Qian-Qian Li, Zhen-Hua Zhang, Qian Xu, Cun-Zhi Liu

**Affiliations:** Acupuncture and Moxibustion Department, Beijing Hospital of Traditional Chinese Medicine Affiliated to Capital Medical University, 23 Meishuguanhou Street, Dongcheng, Beijing 100010, China

## Abstract

Acupuncture stimulation elicits deqi, a composite of unique sensations. According to traditional Chinese medicine (TCM), deqi experienced by patients is often described as *suan* (aching or soreness), *ma* (numbness or tingling), *zhang* (fullness, distention, or pressure), and *zhong* (heaviness) and is felt by the acupuncturists (needle grasping) as tense, tight, and full. It is believed that deqi may be an important variable in the studies of the mechanism and efficacy of acupuncture treatment. In recent years, great efforts have been made to understand deqi, which include a couple of questionnaires to qualify and quantify deqi sensations, neuroimaging studies of deqi and acupuncture, physiological mechanisms of deqi, and the relation between deqi and clinical efficacy. However, many problems need to be resolved, and more researches are required to be made in the future.

## 1. Introduction

Acupuncture is a medical intervention in which needles are used to stimulate certain points generally called acupoints on the body. Traditional Chinese medicine (TCM) indicates that acupuncture stimulation elicits deqi, a composite of unique sensations. It is regarded that the application of acupuncture through stimulating certain acupoints is to activate the *qi* and blood of meridians and collaterals and to regulate the function of internal organs so as to prevent and treat diseases in TCM theory. Therefore, deqi, which literally means “the arrival of vital energy,” is a prerequisite for clinical effects, also an important judgment of the exuberance and decline of meridian *qi* and the prognosis of disease [1]. In addition, it may be of great importance to understand acupuncture mechanisms [2].

In recent years, evocation of deqi has been paid increasing attention in clinical trials of acupuncture, but the physiological mechanisms that produce deqi effect are still not well understood. Few investigators have made explicit efforts to describe deqi from both the patients' and the acupuncturists' perspective and to examine the relationship between deqi and therapeutic effect. Researches in this field focus more on the clinical characterization of the deqi, qualitative and quantitative measurements of deqi, and physiological mechanisms of deqi effect. 

## 2. Characterization of Deqi during Acupuncture Treatment

Deqi is commonly translated as “needle sensation,” sometimes as “arrival of *qi*” or “needling response.” The current view holds that there is no significant difference between them. However, some have different understandings of these three words. Needling sensation is mainly meant subjective feelings and perceived responses of patients and acupuncturists; arrival of *qi* is a healing process, which activates the antipathogenic *qi* to expel the pathogens; the needling response suggests the final aim of acupuncture [3]. Deqi is usually used to describe the subjective sensations felt by the patients during acupuncture treatment, but the view is not shared by all, and some argue that deqi comprises not only the patients' sensations but also the acupuncturists' senses. Furthermore, there are few people suggesting that deqi also includes propagated sensation along meridians and the externally visible physical signs due to acupuncture treatment [4].

### 2.1. Characterization of Deqi Felt by the Patients

Over the past decades, researchers have put more weight on the patients' rather than the acupuncturists' experience during needling. This may be due in part to the rising popularity of new acupuncture modalities such as electroacupuncture [1]. Multiple sensations around the acupoints experienced by the patients are often described as* suan* (aching or soreness), *ma* (numbness or tingling), *zhang* (fullness/distention or pressure), and *zhong *(heaviness) in the literature according to TCM [5]. Besides, pain, which is experienced occasionally, has not been well characterized [6]. Dull pain is considered as deqi and beneficial to treatment, while sharp pain is not deqi and harmful [7]. Patients experience deqi very differently because of conditions of constitution or the therapists' manipulation, such as the direction, angle, and depth of needling [8, 9]. Nevertheless, some studies have shown that the sensations are similar between subjects, irrespective of their constitution, expectation, or cultural background [7, 10]. Recently, a study taking cultural differences into account shows that Chinese patients enjoy deqi experience whereas Americans do not [7]. No significant difference is found in the needling sensations among different acupoints [6]. Deqi sensation appears to be qualitatively and quantitatively different between manual and electrical stimulation. Aching is the most predominant deqi sensation of the former, whereas the latter is tingling [11]. 

### 2.2. Characterization of Deqi Felt by the Acupuncturists

Although the most popular view focuses mainly on patients' sensation, *Huangdi Neijing* (Huangdi's canon of medicine), one of the four great classics in TCM, states that deqi should be felt by the acupuncturists who also need to concentrate in order to hold it [12]. The increased resistance of the needle is felt by the acupuncturists (needle grasping) as tense, tight, and full like “a fish biting onto the bait” [13] or arrival of *qi* like “a bird flying” [14] as described in the ancient literature. Needle grasping is considered to be associated with clinical efficacy although little data is available [15].

### 2.3. Physical Signs due to Acupuncture Treatment

Another important feature of deqi is that it often spreads or radiates from the point of its elicitation, which is called “propagated sensation along meridians” (PSM) or, more commonly, “propagated sensation along channels” (PSC) [16], which is explained as the flow of *qi*. PSC is observed to “jump” between adjacent meridians through geographic information system, suggesting a close connection between PSC and classical meridians [2]. There are not obvious differences between acupoints for the distance of sensation transmission [6]. Sometimes, it may manifest like skin redness, gooseflesh, or localized red or white lines along the meridians of the body surface [4]. 

Although the characterization of deqi is mentioned, respectively, in the previous section, the patients' sensations and the acupuncturists' senses are closely linked. When the acupuncturists feel tense or tight, the patients usually experience soreness, numbness, fullness, or heaviness at the same time. Under the circumstances that the *qi *has not arrived, the patients have no special sensation or response and acupuncturists feel slow, slipping, or empty. It has been vividly described in classical prose named “*biao you fu*” [13]. Now most researchers agree with the explanation of the phenomenon just as the following. Acupuncture through stimulating certain acupoints may contract intraspindle muscle and then produce myoelectricity. Secondary impulse reaching to central brain produces the patients' needling sensation, and the contraction of local muscle fibers through needle body to needle handle causes the deqi sensation of acupuncturists' hands.

## 3. Qualitative and Quantitative Measurements of Deqi

Deqi may be an important variable in studies of the efficacy and mechanism of acupuncture treatment. Some attempts of developing deqi questionnaires (Table 1) have been made to measure deqi sensation. However, there is still no consensus for a method or instrument to qualify and quantify deqi sensation despite efforts towards this goal.

### 3.1. Deqi Questionnaires Discriminating Deqi from Pain

Vincent and colleagues started this work a couple of decades ago. To monitor needling sensations, Vincent et al. [17] condensed the McGill Pain Questionnaire [18] to 20 adjectives describing deqi by applying expert consensus. Park et al. modified the Vincent scale by adding five sensations based on the comprehensive literature review, including both pain and deqi sensations. These sensations primarily came from pain questionnaires and did not focus specifically on deqi [10].

Acupuncture needling evokes two sensations: pain and deqi. Pain is usually caused by penetrating the skin, whereas deqi is possibly caused by stimulating deeper structures at acupoints [19]. Sharp pain is believed to result from inadvertent noxious stimulation rather than deqi, as evidenced by distinct differences in hemodynamic response by fMRI [20]. Therefore, it is important to discriminate deqi from pain. MacPherson and Asghar [8] further examined the Park questionnaire using a “Delphi process” to separate the deqi sensation and pain. Based on a hierarchical cluster analysis, a group of seven sensations was found to be associated with the category of deqi, and a group of nine sensations was associated with the category of acute pain. Later on, the Southampton Needle Sensation Questionnaire (SNSQ), developed by White and colleagues [21], was shown to be a valid, rigorous, soundly grounded, and patient-centered measurement and to enable the discrimination between pain and deqi sensation. Pach et al. [22] tried to create a German version of the SNSQ in order to measure deqi in subjects receiving different forms of acupuncture and to evaluate the translated questionnaire. However, for the language and cultural differences, factor structure of the original questionnaire could not be reproduced with the German version of the SNSQ in an experimental setting. The above-mentioned questionnaires did not involve interviews with patients to ask them to describe what sensations they perceived when they received acupuncture, which appeared to be the major design flaw. 

### 3.2. Deqi Questionnaires with Patients' Interviews

To address the complexity involved in accurately assessing deqi, Kong et al. created a scale entitled “Subjective Acupuncture Sensation Scale (SASS)” in 2005 [23] when launching a study on acupuncture analgesia in 2000. Nine sensations mainly based on the traditional literature were listed on the scale. What is more was that one supplementary row at the end of the nine descriptors was left blank for subjects to describe perceptions in their own words. Using this instrument, it was possible to show significant correlations between the feeling of numbness as well as soreness and the analgesic effect of acupuncture. After deliberating with other acupuncture research groups, Kong et al. modified the SASS to make it useful to a wider range of research projects, which was called “MGH Acupuncture Sensation Scale (MASS)” [1]. The scale included twelve descriptors modified to form a more comprehensive set of sensations, one supplementary row (describing perceptions in their own words), and two supplementaries (“Acupuncture Sensation Spreading Scale” and “Mood Scale”). Yu et al. developed a Chinese version of MASS, namely, Modified MASS-Chinese (C-MMASS), a valid and reliable instrument for the assessment of needle sensations in Hong Kong Chinese people receiving electroacupuncture. “Sharp pain” was removed from C-MMASS [24]. Mao et al. [25] developed a questionnaire and conducted a descriptive survey, including eleven needling sensations, an open-ended question of additional deqi sensations, and a survey of asking about situation of PSC. Five items were specifically designed with response options ranged from “completely disagree” to “completely agree” to capture the patients' attitudes and beliefs about needling sensations. The common characteristics of deqi and its migratory nature could be described through the questionnaire.

### 3.3. Other Scales

Furthermore, Hui et al. explored “deqi composite,” an approach proposed for reducing the complex sensation profile of deqi to a single value, which would facilitate more straightforward comparisons between subjects, acupoints, or stimulation techniques [9]. This index could be used as a covariate in the future exploration of the hemodynamic response of the brain to acupuncture demonstrated by fMRI and its correlation with the efficacy of acupuncture in clinical practice. Kou et al. confirmed that Visual Analogue Scale (VAS) was an objective and reliable way to quantify deqi sensation [26]. The questionnaire was given to subjects to evaluate deqi sensations, including numbness, pressure, heaviness, warmth, and radiating paraesthesia, respectively. A separate VAS to measure their levels of anxiety during the treatment was also included. The results showed that acupuncture significantly induced higher VAS values for numbness, pressure, warmth, and radiating paraesthesia but not for heaviness than the placebo. However, the results were not able to clearly distinguish the deqi sensation over each individual acupoint. 

Though there are several ways to qualify and quantify deqi sensation, an international standard questionnaire is still needed to accurately describe deqi. The questionnaires are needed to be designed more comprehensively, including the acupuncturist senses as well as the physical signs under the condition of a consistent definition of deqi. It might also be necessary to rethink the measurement of deqi with questionnaires. Such a measurement could be used in clinical trials for different diseases, making it possible to use specific sensations to predict the outcome of treatments and strengthen our understanding of acupuncture mechanisms. For the construct which is sensitive to cultural and ethical background, or strong subjectivity of deqi itself, perhaps the idea of an international standard questionnaire has to be rejected before. The development of such a questionnaire might have to go through the whole process. 

## 4. Neuroimaging Studies on Deqi Sensation and Acupuncture Effect

Modern neuroimaging has provided revolutionary tools to monitor the dynamic response of the whole brain to acupuncture with specific regional localization. Functional magnetic resonance imaging (fMRI) and positron emission tomography (PET) studies on acupuncture at commonly used acupoints have demonstrated the limbic system and paralimbic, hypothalamus, and subcortical gray structures as the important components in mediating the acupuncture effects and deqi [23, 27, 28].

Over the past decade, Hui et al. have built a database of fMRI scans of the brain response to acupuncture at multiple acupoints, LI4 (*hegu*), ST36 (*zusanli*), and LV3 (*taichong*) in healthy adults [20, 29, 30]. Their studies showed that acupuncture deqi evoked deactivation of a limbic-paralimbic-neocortical network, which encompassed the limbic system as well as activation of somatosensory brain regions. Importantly, Hui et al. consistently observed distinct patterns of limbic network hemodynamic response in the brain, mainly deactivation in deqi and activation in sharp pain. The findings were consistent with previous reports [8, 21, 31]. Hsieh et al. showed via PET that elicitation of deqi resulted in a significant increase of blood flow in the hypothalamus and insula with an extension to the midbrain when compared with minimal or no stimulation after needle insertion at LI4 [32]. Napadow et al. found that perceptions from acupuncture were preferentially processed by the dorsomedial prefrontal cortex through continuously monitoring rating of acupuncture sensations during fMRI. Deqi fostered acupuncture analgesia through focusing attention and accentuating the bodily awareness which in turn could enhance antinociceptive mechanisms within the central pain network [33]. Lai et al. revealed a significant difference in activated brain areas and brain metabolic changes when deqi was achieved by proper needle manipulation in SJ5 (*waiguan*) using PET in healthy volunteers. These studies mostly focused on healthy subjects, and the cerebral changes in patients who received acupuncture and the pathological conditions were rarely explored [34]. And for all we know, there was a study which showed that the activation of the hypothalamus was more robust in the heroin addicts than that in the healthy subjects during acupuncture. The deqi scores of the heroin addicts were significantly higher than those of the healthy subjects [35].

 Hui et al. observed signal decreases in hypothalamus and nucleus accumbens with acupuncture deqi at both LI4 and ST36. But others reported increasing signals, using fMRI for acupuncture at both ST36 and LI4 [36] and using PET for acupuncture at LI4 [32]. Differences were also found among acupoints, with LI4 showing more prominent response than other commonly used acupoints, which may provide scientific support for why LI4 was frequently employed in clinical practice to a certain extent [37]. In comparison of modulation effect in the limbic-medial prefrontal network, there was a little stronger signal in ST36 than in CV4 (*guanyuan*) by using fMRI, which also indicated acupoint specificity [38]. It was proposed that the magnitude of signal change observed in acupuncture deqi was small, generally less than 1%, compared with the 2–4% activation by visual stimulation or other sensory tasks reported in the literature [39]. The smaller response suggested that acupuncture, unlike noxious insults and pharmacological agents, might act within physiological limits. This could explain in part why acupuncture treatment generally caused fewer side effects than medications, particularly potent analgesics.

## 5. Physiological Mechanisms of Deqi

In combination with techniques, basic scientific studies have begun to elucidate the physiological mechanism that produces deqi effect. In previous studies, researchers [40] have found that stimulation of vessels, nerves, muscles, tendons, and periostea could evoke variable sensations thus producing varying effects in the central nervous system and human body. Predominantly, stimulation of nerve branches produced numbness; stimulation of muscles produced soreness and distention; and stimulation of blood vessels produced pain. It was also demonstrated that many of the deqi sensations were conveyed by different nerve fiber systems without reaching the threshold of overt noxious simulation. Aching, soreness, distension, heaviness, warmth, and dull pain were conveyed by the slower conducting A**δ** and C  fibers, whereas numbness was conveyed by the faster conducting A*β*  fibers in the skin [41, 42]. Deqi was also suggested to relate to activation of high-threshold ergoreceptors in muscle [43]. 

Deqi can help in regulating the blood flow with a certain degree of meridian specificity by using speckle laser blood flow scanning technology [44]. Sandberg et al. indicated that the intensity of deqi resulted in pronounced increase in both skin and muscle blood flows using photoplethysmography [45]. It was also proved that deqi had close correlations with the decrease in blood flow velocity while acupuncture on SP3 (*taibai*) [46]. Irnich et al. performed a trial comparing sham acupuncture to laser acupuncture. The results suggested that deqi may be caused by central processes of awareness rather than the redlight itself provoking deqi sensations directly within the skin [47]. Comparing placebo and deqi acupuncture, it was found that after acupuncture, the former showed a universal increase of transcutaneous CO_2_ emission, while the latter showed a significant increase of transcutaneous CO_2_ emission specifically at acupoints located on the same meridian [48]. It was also observed that the greater acupuncture intensity is, the greater modifications of neurophysiological parameters are [49]. 

## 6. Relation between Deqi and Clinical Efficacy

According to TCM, deqi sensation is believed to be related to clinical efficacy. Manipulation and retaining the needle may strengthen the deqi sensation and improve clinical efficacy in some degree. Now, no studies to our knowledge have systemically investigated the relationship between different aspects of deqi and treatment effects. 

Deqi is suggested to be the main mechanism producing effects of acupuncture [35, 50], for example, by generating a release of spinal and supraspinal beta-endorphins, proinflammatory neuropeptides, and an increase in peripheral circulation [51]. fMRI studies have also found a positive correlation between a subject's psychophysical and hemodynamic responses that strong deqi sensations induce strong deactivation of the limbic system, which result in clinical beneficial effect [20, 30].

However, for the clinical trials, there is still opposite evidence. Enblom et al. found that verum acupuncture, eliciting deqi, was not more effective than sham acupuncture in reducing emesis in cancer patients receiving radiotherapy [52]. White et al. indicated that the presence and intensity of deqi, using the subscale of the Park questionnaire, had no significant influence on the pain relief for the treatment of osteoarthritis of the hip and knee [53]. As we all know, now there is still no strict randomized controlled clinical trial to prove the necessity of deqi to acupuncture. The current conclusion is just based on TCM theory and clinical experience.

Traditional Chinese acupuncture intentionally elicits deqi sensations in patients and regards them as signs of treatment efficacy, but this is not true for all forms of acupuncture. Other styles, such as traditional Japanese acupuncture and wrist-ankle acupuncture, avoid inducing needling sensations in patients. For these forms of acupuncture, treatment effect may be related only to the acupuncturist's perception of deqi or not related to deqi at all—measured entirely in terms of symptom relief.

## 7. Conclusion

Deqi is of great importance to clinical effects and mechanisms of acupuncture treatment, which also need quite a lot of efforts to deeply understand although a few progress has been made. For the subjectivity of deqi, how to understand deqi more scientifically and objectively is more critical. Acupuncture is effective for many diseases, while unclear mechanisms limit its development. Deqi should be further explored in future clinical trials, and more researches are required to understand the underlying mechanisms.

## Figures and Tables

**Table 1:** The common questionnaires in deqi assessment.

Scale	Year	Group	Feature
Vincent questionnaire	1989	Vincent et al. [17]	The sensations primarily coming from pain questionnaires
Park questionnaire	2002	Park et al. [10]	

Macpherson questionnaire	2006	MacPherson and Asghar [8]	Separating the deqi sensations and pain

SNSQ	2008	White et al. [21]	A valid, rigorous, soundly grounded, and patient-centered measurement, enabling the discrimination between pain and deqi

German version of SNSQ	2011	Pach et al. [22]	For the language and cultural differences, the original questionnaire could not be reproduced

SASS	2005	Kong et al. [23]	One supplementary row was left blank for subjects to describe perceptions in their own words

MASS	2007	Kong et al. [1]	Including 12 descriptors, one supplementary row to describe perceptions, and two supplementaries (Acupuncture Sensation Spreading Scale and Mood Scale)

C-MMASS	2012	Yu et al. [24]	Chinese version of the MASS with “sharp pain” removed

Mao questionnaire	2007	Mao et al. [25]	Including 11 needling sensations, an open-ended question of additional deqi sensations, the situation of PSC, and 5 specifically designed items

Deqi composite	2007	Hui et al. [9]	An approach proposed for reducing the complex sensation profile of deqi to a single value

Kou questionnaire	2007	Kou et al. [26]	Evaluating 5 deqi sensations and anxiety using VAS
